# Status Epilepticus and Lateralized Periodic Discharges (LPDs) in Cannabis Withdrawal Syndrome: A Case Report and Review of the Literature

**DOI:** 10.7759/cureus.113823

**Published:** 2026-08-02

**Authors:** Sara Azhar, Hajar Naciri Darai, Fatima Ouchkat, Houyam Tibar, Wafa Regragui

**Affiliations:** 1 Department of Neurology B and Neurogenetics, Specialities Hospital, Ibn Sina Teaching Hospital, Mohammed V University in Rabat, Rabat, MAR; 2 Department of Neurophysiology, Specialities Hospital, Ibn Sina Teaching Hospital, Mohammed V University in Rabat, Rabat, MAR

**Keywords:** cannabis withdrawal syndrome, eeg, epilepsy, periodic lateralized epileptiform discharges, postictal state, status epilepticus

## Abstract

Withdrawal syndrome is relatively common among chronic cannabis users. It generally manifests as psychiatric disorders and, in rare cases, as epileptic seizures. We report the case of a 53-year-old female patient who, following the abrupt cessation of her chronic and daily cannabis use, presented with a tonic-clonic status epilepticus. She was treated with midazolam and phenobarbital. The electroencephalogram (EEG) performed after the cessation of convulsive episodes and the persistence of a state of confusion revealed over a slowed background activity the presence of lateralized periodic discharges (LPDs). Brain imaging, biological workup, and cerebrospinal fluid study ruled out any other causes of secondary epileptic seizures. The patient was placed on benzodiazepine and carbamazepine. The course of the condition was marked by clinical improvement and the disappearance of the LPDs on the follow-up EEG. Our case highlights the possibility of serious neurological manifestations, such as status epilepticus, following abrupt cannabis withdrawal, associated with the presence of lateralized periodic discharges (LPDs) on the EEG. This association is linked to a high risk of recurrent epileptic seizures and therefore requires the initiation of anticonvulsant treatment.

## Introduction

Withdrawal syndrome is relatively common among individuals with cannabis dependence. Its prevalence is estimated at over 50% [1]. It typically appears within 24 to 72 hours (up to one month) of stopping cannabis use and usually resolves within 4 to 14 days [2].

It is mainly characterized by psychiatric symptoms such as anxiety, irritability, aggressive behaviour, and loss of appetite. It can also lead to sleep disturbances and physical symptoms such as abdominal pain, fever, or tremors [1,2]. Rarely, other neurological manifestations have been documented in the literature such as epileptic seizures [3].

We report the case of a patient who presented with status epilepticus complicated by prolonged post-ictal confusion, following the abrupt cessation of her daily cannabis use, and in whom the electroencephalogram (EEG) revealed lateralized periodic discharges (LPDs). These are EEG patterns characterized by recurrent sharp waves, spikes, or spike-wave complexes occurring at regular or near-regular intervals [4].

## Case presentation

A 53-year-old female patient with a history of hypertension, severe obesity, and occasional smoking of tobacco and cannabis daily. Her cannabis use was recurrent during every day (without a precise dose) and for about 20 years. No alcohol, other drugs, or psychotropic medications use was reported. She was admitted to the emergency department following a tonic-clonic status epilepticus (SE) episode that lasted about 30 minutes and which occurred in the absence of fever and with no focal symptoms observed at the clinical examination. One week prior to admission, the patient abruptly stopped using cannabis following hepatic colic associated with gallstones. Two days after stopping cannabis, she presented with psychiatric symptoms consisting of irritability associated with significant anxiety.

**Figure 1:**
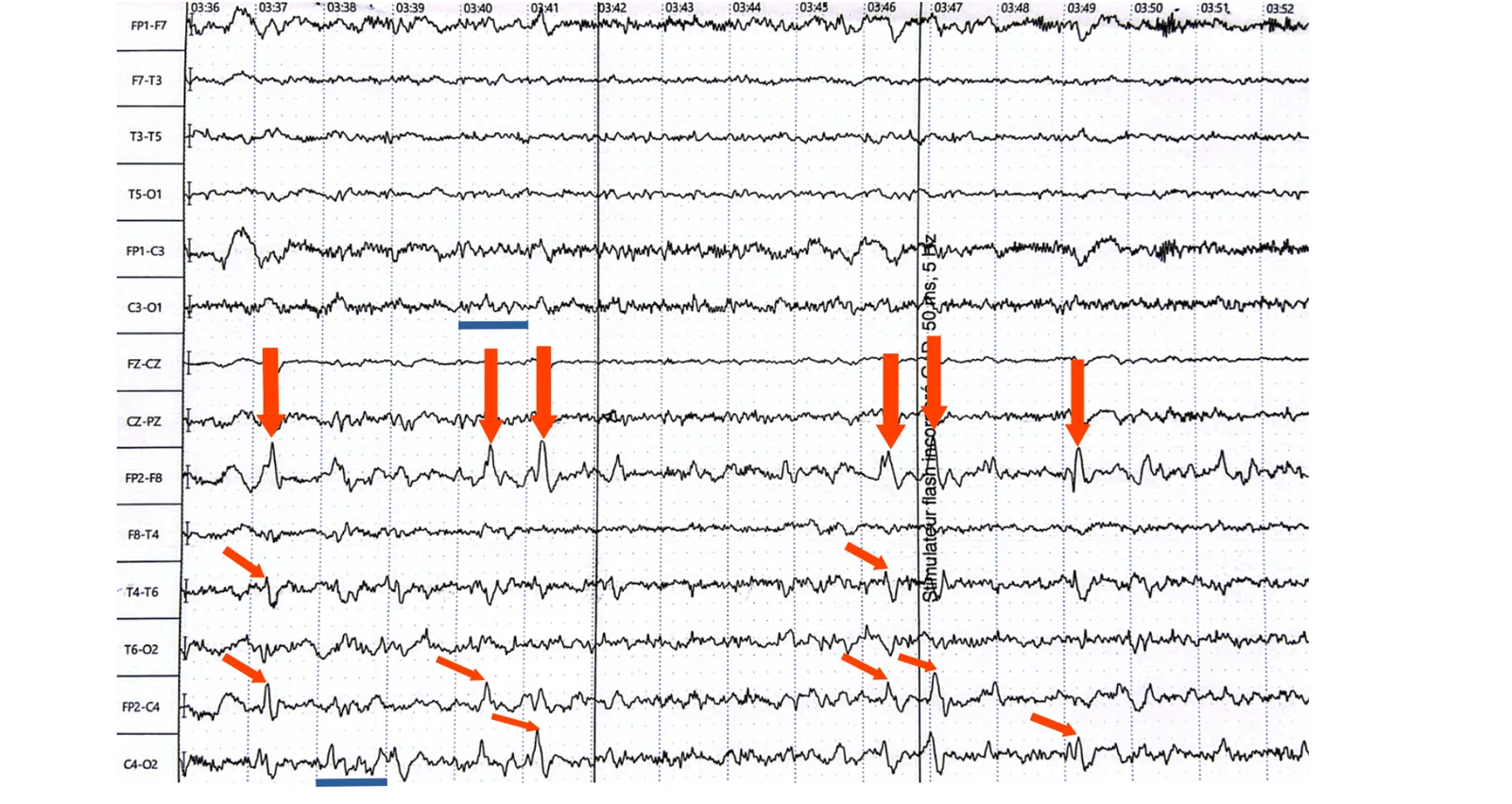
EEG capturing LPDs EEG in a longitudinal configuration (10 microvolts/millimeter) showed a slowed background activity at 5 Hz (seen in the blue lines) and the presence of paroxysmal pseudo-periodic right hemispheric sharp waves: LPDs (seen in the red arrows)

In the emergency room, the SE was controlled after the administration of two intramuscular midazolam loading doses of 0.2 mg/kg and intravenous phenobarbital as a loading dose of 15 mg/kg. However, upon waking, the patient remained confused for three days. The EEG performed three hours after the seizures had ceased showed a general slowing of background activity at 5 Hz with the presence of paroxysmal abnormalities in the form of pseudo-periodic right-hemispheric sharp waves corresponding to LPDs (Figure 1). Continuous EEG was not available for our patient.

Brain magnetic resonance imaging (MRI) was normal, which eliminates posterior reversible encephalopathy syndrome (PRES). The metabolic workup, including serum electrolytes, liver and renal function tests, and vitamin B12 and B9 levels, as well as the infectious workup, including C-reactive protein (CRP), erythrocyte sedimentation rate (ESR), and cerebrospinal fluid (CSF) cytological, biochemical, and microbiological analyses, was unremarkable. Screening for toxins in the urine revealed the presence of cannabinoids (Table 1). Alcohol screening test was not carried out.

**Table 1:** Results of the urine toxicology screening

Urine analysis	Results
Urine toxicology screening	Positive for cannabinoids; negative for other tested substances, including amphetamine, cocaine, benzodiazepines

The patient was placed on carbamazepine and benzodiazepine, resulting in improved alertness, restoration of background activity, and resolution of the LPDs. Carbamazepine was maintained, and no epileptic seizures have occurred since the patient’s SE, with a follow-up of two and a half years.

## Discussion

Our patient presented with first-time SE following the abrupt cessation of her chronic and daily cannabis use. No history of alcohol consumption was reported. Although cannabis withdrawal is known to cause psychiatric symptoms, it can more rarely lead to neurological symptoms, including epileptic seizures [3,5].

Cannabis is a complex plant-derived substance containing several hundred chemical constituents, including more than 100 identified cannabinoids. The principal psychoactive component is Δ9-tetrahydrocannabinol (Δ9-THC), which is responsible for the psychotropic effects of cannabis, whereas cannabidiol (CBD) is the second major cannabinoid. The chemical composition of cannabis products currently available varies according to the plant's genetic background, cultivation conditions, and breeding practices [6-8]. CBD has demonstrated anticonvulsant efficacy in several forms of drug-resistant epilepsy, primarily by modulating neuronal excitability through GPR55 receptor antagonism, regulating voltage-gated ion channels (particularly sodium and calcium channels), and attenuating neuroinflammation. In contrast, THC acts as a partial agonist of cannabinoid receptor type 1 (CB1), modulating the presynaptic release of both excitatory and inhibitory neurotransmitters. Although the anticonvulsant effects of THC have been reported in experimental models, clinical evidence remains limited [9,10]. Abrupt cessation of chronic cannabis use may lead to the loss of inhibition of excitatory neuronal activity, thereby potentially precipitating epileptic seizures [5].

The half-life of CBD is 60 hours, and withdrawal symptoms typically peak between 2 and 6 days [2]. The half-life of THC is between 20 hours and a few days. Our patient presented with psychiatric symptoms 48 hours after stopping cannabis and neurological symptoms 1 week later. Indeed, symptoms may appear late, up to one month later, particularly in obese individuals, such as our patient, due to the high lipophilicity of CBD and THC, and consequently their distribution over a larger volume, resulting in prolonged release [11,12].

A comprehensive assessment that ruled out any other causes of secondary epileptic seizures (structural and metabolic) and the psychiatric symptoms that had preceded the seizures made it possible to probably associate the SE with cannabis withdrawal. However, a coincidental new-onset SE in a middle-aged patient with vascular risk factors (hypertension, severe obesity), which might precede detectable MRI lesions, and potential missed toxic exposures due to limits of standard urine toxicologic screen and the non-availability of alcohol blood levels are possible.

In the emergency department, our patient was treated with midazolam and phenobarbital, with a good clinical response. The convulsive episodes ceased, and her level of consciousness improved, but she remained confused for three days. An EEG was performed, revealing right hemispheric sharp waves in the form of paroxysmal pseudo-periodic discharges corresponding to LPDs. These are unusual electrophysiological patterns characterized by spikes, spike-waves, or sharp waves, which are periodic on at least one lead. They are subdivided into three groups: lateralized to a single hemisphere, which is the case of our patient, focal, or predominant on one side with contralateral spread [4].

From a pathophysiological perspective, LPDs reflect a change in the excitatory transmission of neurons following recent brain damage. This damage may be lesional in nature, in the case of vascular, infectious, or inflammatory origin. It may also be non-lesional, linked to metabolic disorders, drug or alcohol intoxication, and, more rarely, withdrawal from a toxic substance: cannabis for our patient. The lateralization of these electrical abnormalities does not necessarily indicate an ipsilateral lesion [4].

Clinically, the manifestations associated with LPDs include epileptic seizures in 80% of cases [4,13], predominantly focal seizures, particularly motor seizures. Other critical focal manifestations have been reported in sporadic cases, including behavioral arrest, oral automatisms, and complex visual or auditory hallucinations. Cases of SE and post-critical confusional states have also been reported [4].

During SE, the EEG typically shows evolving rhythmic ictal activity, characterized by continuous or quasi-continuous epileptiform discharges, with a progressive increase in their frequency, amplitude, and spatial spread. With treatment or as the condition progresses spontaneously, these patterns may give way to activities belonging to the ictal-interictal continuum, notably LPDs. These can be classified into five classes. Low-grade LPDs (classes one and two) (Table 2) are most often associated with post-ictal activity and are frequently observed during the recovery phase of SE or during the post-critical confusional state, reflecting underlying cerebral injury. High-grade classes or LPDs plus (classes four and five) (Table 2), associated with rhythmic activity, retain a high epileptic value and are closely linked to the persistence of ictal activity, suggesting either uncontrolled SE or non-convulsive SE. The intermediate class (class three) (Table 2) lies on the ictal-interictal continuum and reflects a transition zone, sometimes making it difficult to distinguish between persistent ictal activity and the post-ictal phase [4,14,15]. Our patient’s EEG therefore corresponded to class one LPDs.

**Table 2:** Classification of LPDs The electrophysiological characteristics of different classes of LPDs (adapted from Reiher’s classification) [4] LPD: lateralized periodic discharge

Classes of LPDs				
Proper LPDs (LPDs without rhythmic discharges)			LPDs plus (LPDs associated with rhythmic discharges)	
Class 1	Class	Class 3	Class 4	Class 5
LPDs appear at variable and prolonged intervals throughout the whole recording.	LPDs appear at regular intervals in an intermittent manner.	LPDs appear at regular intervals throughout the whole recording	LPDs with rhythmic discharges that are brief (≤ 1 second)	LPDs with rhythmic discharges that are prolonged (>1 second)
Low grade: Post-ictal patterns with lower ictal significance		The intermediate class	High grade: Persistence of ictal activity, high epileptic value	

On brain MRI, lesions observed during the ictal phase appear as early hyperperfusion on arterial spin labeling (ASL) sequences and disappear a few hours after the seizures have been brought under control; this is sometimes accompanied by a hyperintensity on diffusion-weighted imaging with reduced apparent diffusion coefficient (ADC), reflecting cytotoxic damage linked to neuronal hyperactivity. During the post-critical phase, MRI will typically show hypoperfusion or normal perfusion with diffusion sequences that return to normal 3 to 10 days after the cessation of seizures [16,17].

For our patient, an EEG and a brain MRI were performed a few hours after the seizure activity had ceased and whilst she remained in a state of confusion. Her brain MRI, including diffusion sequences (ASL sequences were not available), was normal, which did not rule out subclinical SE. However, her EEG had shown class one LPDs, which was consistent with a post-ictal state. This state generally lasts less than 24 hours but can persist for up to three days in cases of severe epileptic seizures [18], as was the case of our patient.

The return to a normal state of alertness often precedes the resolution of LPDs, which can take up to 10 days [17]. This suggests an electroclinical dissociation. This phenomenon can be explained by the persistence of focal cortical hyperexcitability despite the restoration of the thalamocortical networks involved in consciousness. Thus, LPDs should not be interpreted as a direct marker of impaired consciousness, but rather as an indicator of residual pathological cortical activity within the ictal-interictal continuum. The follow-up EEG performed one month later for our patient was normal, confirming the restoration of normal neuronal activity.

The association of LPDs with observable seizures, including status epilepticus for our patient, indicates substantial cortical hyperexcitability and a heightened risk of early seizure recurrence. An antiseizure medication is generally recommended, but duration should be tailored based on the underlying etiology, imaging, EEG evolution, and clinical course [13,19]. Since our patient’s SE, she remained seizure-free with a follow-up of two and a half years.

## Conclusions

This case suggests that abrupt cessation of chronic cannabis use may, in rare instances, be temporally associated with status epilepticus and the transient appearance of lateralized periodic discharges (LPDs) on EEG. Their presence increases the risk of recurrent epileptic seizures. Treatment and follow-up should be individualized according to the underlying cause, seizure recurrence risk, and EEG evolution.
